# Fabrication of Water-Compatible Molecularly Imprinted Resin in a Hydrophilic Deep Eutectic Solvent for the Determination and Purification of Quinolones in Wastewaters

**DOI:** 10.3390/polym11050871

**Published:** 2019-05-13

**Authors:** Weiyang Tang, Kyung Ho Row

**Affiliations:** Department of Chemistry and Chemical Engineering, Inha University, Incheon 402-701, Korea; tangweiyang123@126.com

**Keywords:** molecularly imprinted resin, deep eutectic solvent, hydrophilicity

## Abstract

A novel water-compatible molecularly imprinted resin was prepared in a green solvent deep eutectic solvent (DES). Resorcinol and melamine, as functional monomers with an abundant hydrophilic group, such as –OH, –NH_2_ and –NH–, were introduced into the molecularly imprinted resin (MIR). Three DESs (choline chloride-ethylene glycol, tetramethylammonium bromide-ethylene glycol and tetramethylammonium chloride-ethylene glycol) were used to synthesize the molecularly imprinted resin and the resulting deep eutectic solvent-based molecularly imprinted resins were characterized by particle size analysis, elemental analysis, scanning electron microscopy, Fourier transform infrared spectroscopy and thermogravimetric analysis. The resulting deep eutectic solvent-based molecularly imprinted resins were then applied to the adsorption of quinolones (ofloxacin) in water. The adsorption process of deep eutectic solvent-based molecularly imprinted resin followed the static adsorption model, Langmuir isotherm (*R*^2^ ≥ 0.9618) and kinetic model pseudo-second-order (*R*^2^ > 0.9814). The highest theory adsorption ability of the three kinds of deep eutectic solvent-based molecularly imprinted resins was more than 23.79 mg/g. The choline chloride-ethylene glycol-based MIR was applied to solid-phase extraction for the determination and purification of quinolones (e.g., ciprofloxacin and ofloxacin). The detection limit of deep eutectic solvent-based molecularly imprinted resin-solid-phase extraction method was less than 0.018 mg/L. The recoveries of the deep eutectic solvent-based molecularly imprinted resin-solid-phase extraction method at three spiked levels were 88.7–94.5%, with a relative standard deviation of ≤4.8%. The novel deep eutectic solvent-based molecularly imprinted resin-solid-phase extraction method is a simple, selective and accurate pre-treatment method and can be used to determine the quinolones in environmental water.

## 1. Introduction

Recently, molecularly imprinted polymers (MIPs) have been adopted as a kind of tailor-made receptor with specific molecular recognition sites in terms of the size, shape and functional groups. The main principles of the molecularly imprinted technique (MIT) are based on incorporating functional monomers with a template molecule to form a pre-complex via covalent or non-covalent bonds [1,2,3,4]. Subsequent removal of the template results in the specific recognition cavities that act as molecular recognitions sites to achieve specific binding of the target molecule. With the advantages of specific recognition ability, good stability and uncomplicated synthesis process, MIPs have attracted considerable attention in many fields such as purification and separation [2,3,4,5,6,7,8,9], chemo-sensing [10,11] catalysis [12,13,14] and drug delivery. Currently, most MIPs are based on non-covalent mechanisms and were synthesized in non-polar or less polar solvents by the interaction of hydrogen bonding between the functional monomer and template molecule; they are normally only compatible with non-aqueous solutions. On the other hand, water is the most used matrix in environmental and biological samples. The poor compatibility and molecule-recognition ability of MIPs in the aqueous phase considerably limit the application of MIPs [15]. Furthermore, the large amounts of organic solvents used in the synthesis of MIP are not in accordance with the idea of green and sustainable chemistry. Hence, the development of aqueous-compatible imprinted polymers and the utilization of eco-friendly solvents in the preparation process are imperative.

Exploration of hydrophilic imprinted polymers with excellent molecular recognition ability in aqueous environments attracts considerable attention. The existence of hydrophobic groups on MIPs is considered the main reason for its incompatibility with aqueous environments. Therefore, the use of the hydrophilic monomers was considered to be an effective solution to avoid hydrophobic groups [16]. Hydrophilic resins are prepared with a range of hydrophilic monomers that can be combined with the molecular imprinting technique to produce the molecularly imprinted resin (MIR). Resorcinol-formaldehyde resin is a common hydrophilic resin that contains abundant –OH groups and exhibits excellent separation performance on metal ions [17,18,19]. Melamine-formaldehyde resin has adequate primary/secondary amino groups and good thermal stability [20,21]. Therefore, a combination of the advantages of melamine and resorcinol, in preparing the hydrophilic resin can result in enough hydrophilic groups binding with the target molecules [22]. On the other hand, conventional hydrophilic solvents (such as methanol, ethanol, etc.) can affect the formation of hydrogen bonds between the template and functional monomer during MIR synthesis.

Abbott et al. [23] proposed a class of liquids called deep eutectic solvents (DES) to overcome the disadvantages of conventional aqueous media. DES are generally composed of a hydrogen-bond acceptor (HBA) and hydrogen-bond donor (HBD), which are capable of self-association by hydrogen-bond interaction. A comparison with organic solvents revealed DESs to be more eco-friendly because of their low toxicity and negligible vapor pressure. In addition, the excellent thermal stability, non-flammability and solubility of DESs can be applied to chemical synthesis, extraction and separation [24,25]. In this protocol, the hydrophilic DES was selected as the solvent medium to prepare the DES-based MIR (DES-MIR). This process not only provided the aqueous environment for the preparation of the MIR but it also enhanced the affinity of MIR toward the template molecule. Generally, the hydrogen-bond is the main interaction between the template molecule and functional monomers during the imprinting process. On the other hand, the hydrogen-bond interaction is ruptured easily in conventional aqueous media because of the competition for functional monomers between the template molecule and solvent molecules [26]. DESs, as solvent media, provided an extra ionic interaction that is theoretically stronger than a hydrogen-bond. This characteristic makes it more resistant to the interference of conventional solvent molecules during the process of imprinting and recognition toward analytes.

In this study, a new water-compatible MIR was synthesized in DES solvent media with hydrophilic resorcinol and melamine monomers and formaldehyde cross-linker, which introduced abundant hydrophilic groups into the resin structure. Three types of hydrophilic DESs (choline chloride-ethylene glycol, tetramethylammonium bromide-ethylene glycol and tetramethylammonium chloride-ethylene glycol) were used as green media to enhance the affinity of the MIR to the target in aqueous media. The resulting DES-MIR was applied as adsorbent in solid-phase extraction (SPE) to recognize the quinolones (e.g., ciprofloxacin and ofloxacin) in wastewater. Quinolones are widely used as therapeutic and prophylactic antimicrobial agents in animal husbandry and aquaculture (seafood industry), which is reported by other research groups [24,27]. The widespread use of quinolones in seafood industry has resulted in the potential risk of its residues in water and the development of resistant bacterial strains. The DES-MIR showed excellent compatibility with water and specific molecule recognition ability with higher recoveries than common MIR.

## 2. Experimental

### 2.1. Chemicals and Chromatography Instruments

Choline chloride (ChCl, 98%), tetramethylammonium chloride (TMAC, 99%) tetramethylammonium bromide (TMAB, 99%) and ethylene glycol (EG, 98%) were purchased from Sigma-Aldrich Co, Ltd. (St Louis, MO, United Stated). Ofloxacin (OFL, 99%), ciprofloxacin (CIP, 99%) and melamine monomer (98%) were supplied by Tokyo Chemical Industry Co., Ltd. (Tokyo, Japan). Resorcinol (99%), formaldehyde solution (37%), trifluoroacetic acid (TFA, 99%), methanol (MeOH), acetic acid (HAc), ethyl acetate (EtOAc) and acetonitrile (ACN) were acquired from Duksan Pure Chemical Co., Ltd. (Ansan, Korea). Ultrapure water was used in all experiments. The details of high-performance liquid chromatography (HPLC) instruments and conditions were added to the Supplementary Materials.

### 2.2. Synthesis of Hydrophilic DES and DES-Based MIR

The three hydrophilic DESs were prepared using the same synthetic method reported elsewhere [26]. The two components, HBA and HBD, were mixed into a stand-up flask with stirring at 300 rpm and 80 °C for 2 h. The HBA and HBD component mixtures transformed into a homogeneous liquid with no observed solid. Table 1 lists the components of the DESs (DES-1: ChCl-EG; DES-2: TMAB-EG; DES-3: TMAC-EG).

DES-MIR was synthesized by suspension polymerization and prepared using a slight modification of the methodology reported elsewhere [22]. The details of the DES-MIR synthesis step were added to the supplementary materials. The non-imprinted DES-based resin (DES-NIR) and common molecular imprinted resin (MIR, without DES) were synthesized in an identical manner to DES-MIR, except for the addition of the template and DES. Table 2 lists the components of the materials.

### 2.3. Characterization of DES and DES-MIR

The functional groups details in DES, DES-MIR and MIR were characterized by Fourier transform infrared spectroscopy (FTIR, Vertex 80 V, Bruker, Billerica, MA, USA) using the KBr pellet technique between 4000–400 cm^−1^ at a scan rate of 20 scans/min. The morphology of the materials was examined by scanning electron microscopy (SEM, Hitachi S-4200, Hitachi, Toronto, ON, Canada). The particle size distributions of DES-MIR and MIR were analyzed using a Mastersizer 2000 instrument (Malvern Panalytical, Malvern, UK). The elemental content was determined using an elemental analyzer, EA1112 (Thermo Fisher, Waltham, MA, USA). Thermogravimetric analyses (TGA) were carried on a thermo-microbalance (TG 209 F3, Netzsch, Selb, Germany).

### 2.4. Adsorption Behavior of OFL on the Hydrophilic Resin

In the static adsorption experiment, 10 mg of hydrophilic resin (DES-1-MIR, DES-2-MIR, DES-3-MIR and MIR, respectively) was added in a round bottom flask containing 5 mL of MeOH solutions with 10–200 µg/mL OFL at 25 °C for 2 h and separated by centrifugation at 4000 rpm for 10 min.

A dynamic adsorption test was conducted in parallel as the following descriptions. A 10 mg hydrophilic resin (DES^1^-MIR, DES^2^-MIR, DES^3^-MIR or MIR) was used as an adsorbent to evaluate the performance in 5 mL of the 100 µg/mL OFL solutions with mechanically shaking for different times (5–200 min, respectively). The residual concentration of the OFL was analyzed by HPLC.

The equilibrium adsorption quantity (*Q_e_*) and temporal adsorption quantity (*Q_t_*) were calculated using the following equations:

(1) $$Q_{e}=\frac{(C_{0}-C_{e})\times V}{W}$$

(2) $$Q_{t}=\frac{(C_{0}-C_{t})\times V}{W}$$

where *V* is the volume of the solution and *W* is the mass of the polymer powder. *C_0_*, *C_e_* and *C_t_* are the initial, equilibrium and temporal concentration, respectively.

### 2.5. DES-MIR-Based SPE for Environmental Water

The sample (wastewater) was collected from a local seafood market in Incheon. The wastewater sample was concentrated to dryness at 50 °C and then reconstituted with 1 mL of MeOH for further SPE procedures. First, a 200 mg sample of the different resin particles were packed in an empty SPE cartridge and the frits were placed at the lower and upper ends to avoid polymers loss. The particle-packed cartridge was pretreated with 2 mL of MeOH and water prior to extraction. Subsequently, 1 mL of the sample solution was loaded into the SPE cartridge, washed and eluted with 2 mL of water and 4 mL of ACN-ammonia (95:5, *v*/*v*). Subsequently, the eluent was evaporated to dryness under a gentle N_2_ stream and was reconstituted with 0.1 mL of the mobile phase for HPLC analysis.

## 3. Results and Discussion

### 3.1. Synthesis of DES and DES-Based Hydrophilic MIR

**Figure 1:**
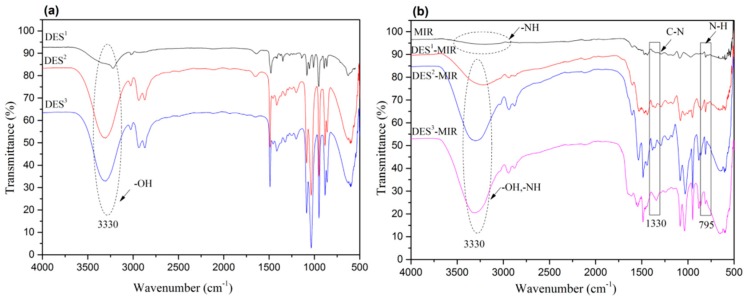
(**a**) Fourier transform infrared (FTIR) spectra of DESs (DES^1^, DES^2^ and DES^3^) and (**b**) DES-molecularly imprinted resin (MIR) (DES^1^-MIR, DES^2^-MIR and DES^3^-MIR).

First, one of the three types of HBAs, (ChCl, TMAC and TMAB) and one HBD (EG) was combined to prepare three different hydrophilic DESs. In this experiment, the structures of DES^1^, DES^2^ and DES^3^ were characterized by FTIR spectroscopy, as shown in Figure 1a. FTIR spectroscopy revealed a peak for the hydroxyl stretching vibration at 3330 cm^−1^, which was attributed to the existence of EG. This shows that all the DESs can provide a sufficient number of hydrophilic structure groups in the synthesis of the DES-MIR process.

**Figure 2:**
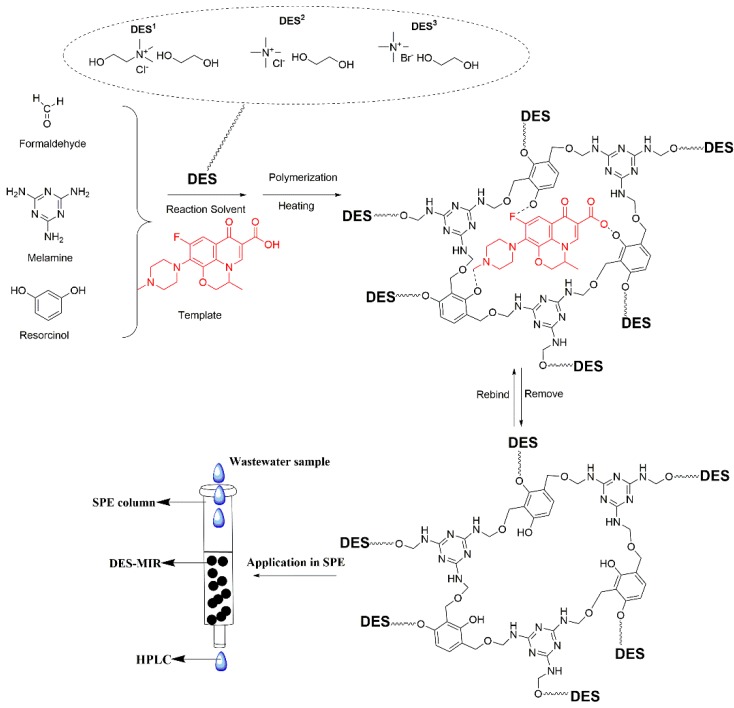
Schematic representation of the fabrication of the DES-MIR and application in solid-phase extraction (SPE).

Three types of DES-MIRs (DES^1^-MIR, DES^2^-MIR and DES^3^-MIR) were prepared with the dual functionality of the resorcinol and melamine monomers incorporated into the MIR. To enhance the hydrogen-bond ability between the template and functional monomer during MIR synthesis, three types of DESs were introduced to the polymerization process as the reaction media. Figure 2 presents a schematic diagram of DES-MIR formation.

### 3.2. Characterization of DES-MIR

The FTIR spectra of the synthesized DES-MIRs in Figure 1b revealed some mutual hydrophilic groups on the polymer surface, such as –OH and –NH–. A comparison with MIR, showed that DES^1^-MIR, DES^2^-MIR and DES^3^-MIR had a strong FTIR peak at 3330 cm^−1^, which was assigned to the –OH stretching vibration. This confirmed that DES^1^, DES^2^ and DES^3^ had been entrapped into the MIR. Furthermore, the two medium-intensity peaks at 1330 cm^−1^ and 795 cm^−1^ were assigned to the C-N stretching in the triazine ring and the –N–H out-of-plane bending in melamine, respectively, which indicate the presence of melamine within the DES-MIR [22]. The N, Br and Cl contents of the materials also confirmed the successful impregnation of DES, which means the DESs had been combined with MIR, as listed in Table S1.

**Figure 3:**
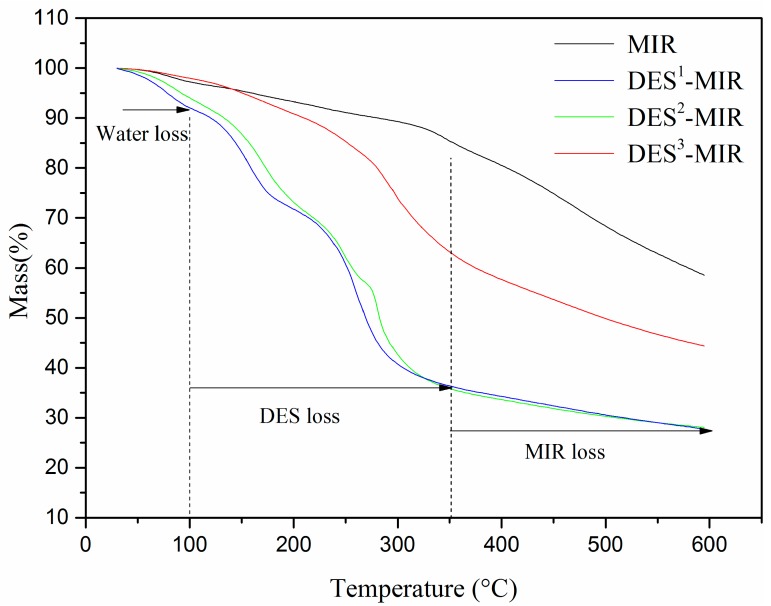
Thermogravimetric (TGA) curves of the MIR and DES-MIR (DES^1^-MIR, DES^2^-MIR and DES^3^-MIR).

TGA was performed to confirm the successful entrapment of DES with MIR (Figure 3). The results revealed a steep weight loss for all the adsorbents (MIR, DES^1^-MIR, DES^2^-MIR and DES^3^-MIR due to the loss of moisture within 100 °C. As the temperature was increased to 350 °C, DES^1^-MIR, DES^2^-MIR and DES^3^-MIR began to degrade from 91.9 to 36.4%, 93.9 to 35.5% and 97.7 to 63.1%, respectively. On the other hand, the mass of MIR with this temperature range did not show an obvious decrease (97.5 to 85.4%). These performances can be attributed to the existence of DES on the surface of the MIR due to the interactions between DES and functional monomers.

**Figure 4:**
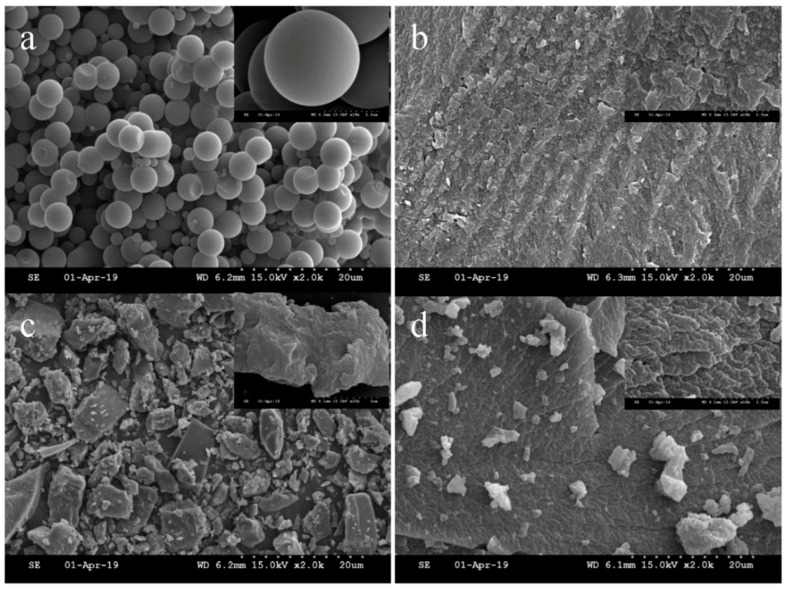
Field emission scanning electron microscopy (FE-SEM) images of (**a**) MIR, (**b**) DES^1^-MIR, (**c**) DES^2^-MIR and (**d**) DES^3^-MIR (magnification: 2.0k× and (inset) 18k×).

The morphology of DES^1^-MIR, DES^2^-MIR, DES^3^-MIR and MIR was analyzed by SEM (Figure 4). The conventional MIR was a microsphere with a smooth surface and the DES-based MIR had a relatively rough surface. SEM images of these DES^1^-MIR, DES^2^-MIR and DES^3^-MIR materials revealed a similar surface morphology: a rough and porous structure. This rough surface may provide more specific recognition sites for the target.

**Figure 5:**
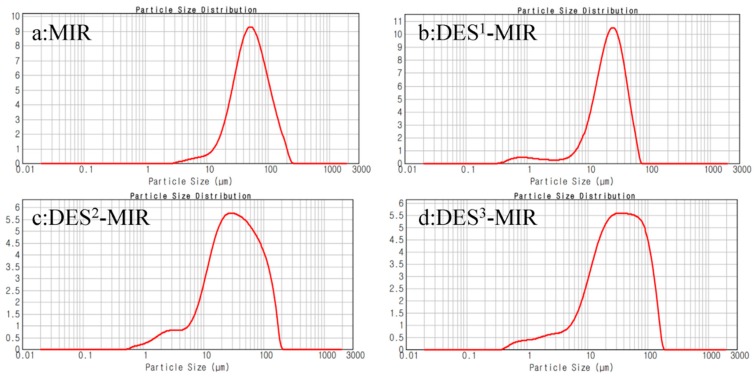
Particle size distribution of (**a**) MIR, (**b**) DES^1^-MIR, (**c**) DES^2^-MIR and (**d**) DES^3^-MIR.

Figure 5 shows the particle size distribution of MIR, DES^1^-MIR, DES^2^-MIR and DES^3^-MIR. Compared to the conventional MIR (particle size *D*(0.5): 51.798 µm), the DES^1^-MIR (particle size *D*(0.5): 22.584 µm), DES^2^-MIR(particle size *D*(0.5): 31.706 µm) and DES^3^-MIR (particle size *D*(0.5): 31.847 µm) had smaller particles. Because of the weak dispersion of the monomer agent (resorcinol and melamine) in the traditional solution, the hydrophilic resin would aggregate, resulting in a larger particle size. DESs as a green solvent can enhance the particle dispersion effect, resulting in smaller particles, which could enhance the adsorption efficiency and increase the affinity toward the target analytes in the aqueous solution.

### 3.3. Hydrophilic Performance of DES-MIR

**Figure 6:**
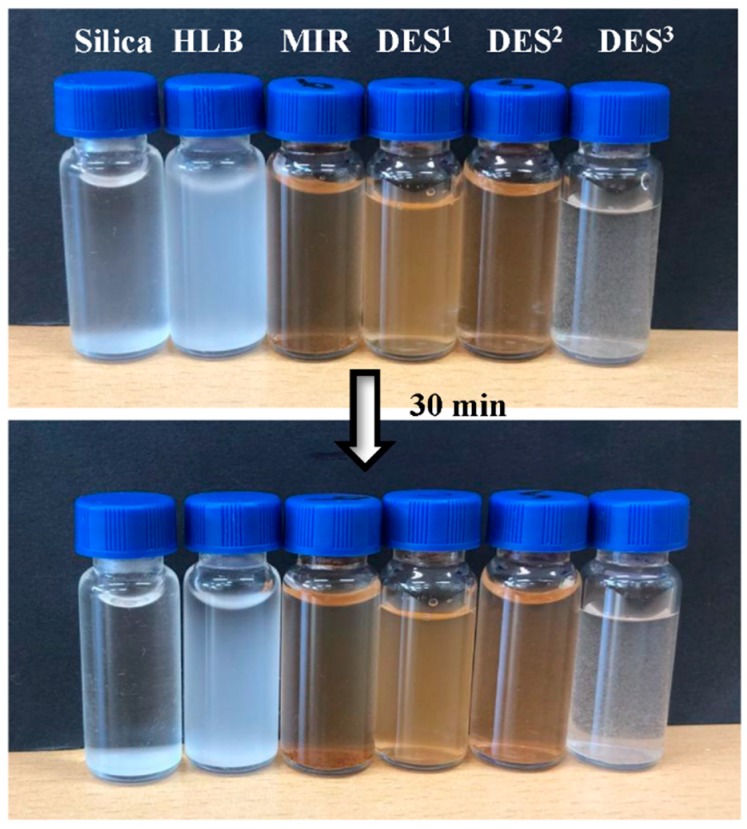
Hydrophilicity performance of the different adsorbents.

To assess the hydrophilic behavior of the synthesized DES-MIR, the dispersion stability of different commercial sorbents, such as silica and HLB, was compared with MIR and DES-MIR. Typically, all the sorbents were dispersed ultrasonically in water with a concentration of 10 mg/mL. Figure 6 presents images of these sorbents. As observed with silica, HLB, MIR, DES^1^-MIR, DES^2^-MIR and DES^3^-MIR could initially be dispersed homogeneously in water. After 30 min, most of the silica, HLB and partial MIR settled to the bottom of the bottle. In contrast, DES^1^-MIR, DES^2^-MIR and DES^3^-MIR still exhibited excellent dispersion ability in water due to the existence of DESs. This shows that DES, as the reaction media, plays an important role in enhancing the compatibility with water.

### 3.4. Static Adsorption and Dynamic Adsorption

**Figure 7:**
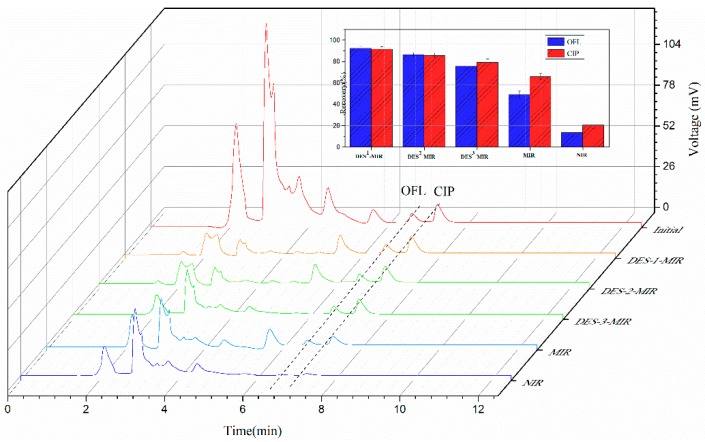
Chromatograms and recoveries of different adsorbents including (NIR, MIR, DES^1^-MIR, DES^2^-MIR and DES^3^-MIR).

To assess the static adsorption performance of OFL over DES-MIRs, three types of adsorption isotherms models, such as Langmuir, Freundlich and Scatchard, were fitted to the isotherm adsorption data. The equations of the three models are as follows and the corresponding linear plots and Figure 7 and Table 3 show the isotherm parameters for the different models. Compared with Freundlich and Scatchard models, the Langmuir isotherm showed better correlation (*R*^2^ ≥ 0.9618) on different DES-MIR adsorbents. The maximum adsorption capacities of the resin adsorbents were estimated from the Langmuir plots. All the DES-MIRs showed higher *Q*_max_ values (>23.79 mg/g) than the conventional MIR (*Q*_max_ = 15.33 mg/g). This difference in adsorption capacity between the DES-MIR and MIR must be due to the adsorption functionality introduced by the DESs within the MIR. Furthermore, the different DESs also showed different adsorption capacities. In this case, DES^1^ had the highest adsorption capacity (*Q*_max_ = 32.92 mg/g) because of its better dispersive capacity than DES^2^ and DES^3^. Consequently, all further adsorption studies were carried out using DES^1^.

Figure 7 shows the dynamic adsorption behavior of DES^1^-MIR and MIR. A comparison with the pristine MIR revealed the DES-impregnated MIR (DES^1^-MIR) to have rapid mass transform efficiency and reach adsorption equilibrium after 100 min. The adsorption data of OFL were fitted using pseudo-first-order and pseudo-second-order kinetic models onto DES^1^-MIR and MIR. The adsorbents showed better compliance with the pseudo-second-order kinetic model (*R*^2^ ≥ 0.9814) than with the pseudo-first-order model (*R*^2^ ≥ 0.8689). Table 4 lists the corresponding kinetic constants and correlation coefficients.

### 3.5. Validation of the DES^1^-MIR-SPE in HPLC

The range of linearity, limits of detection (LOD), limits of quantification (LOQ) and recovery of the DES^1^-MIR-based SPE method were assessed, as listed in Table S2. The calibration curves of the quinolones (OFL and CIP) were constructed with five spiked levels within the range of 0.1–100 μg/mL with good coefficients (*R*^2^ ≥ 0.9989). The LOD and LOQ of OFL and CIP were 0.012 μg/mL & 0.040 μg/mL and 0.018 μg/mL & 0.060 μg/mL, respectively. The intra-day and inter-day precision in this method were obtained by spiking the wastewater samples at three levels (1, 10 and 100). The recoveries of OFL and CIP ranged from 91.7 to 94.5% and 88.7 to 94.4% with the RSDs less than 3.3 and 4.6, respectively. This shows that DES^1^-MIR-SPE is a sensitive and accurate analysis method (Table S3). Furthermore, a comparison of present method with previously reported method was indicated in Table S4. The results revealed DES^1^-MIR-SPE method has the excellent analysis performance to analyze OFL and CIP in wastewater samples with good recoveries.

### 3.6. Application of DES-MIR in SPE for the Determination of Quinolones in Wastewater

The feasibility of the DES^1^-MIR-based SPE method was assessed by the purification and extraction OFL and CIP in a wastewater sample. Different types of environmental water samples were obtained and analyzed using the DES^1^-MIR-SPE method, as listed in Table S5. One of the samples obtained from a local seafood market was found to contain a trace amount of OFL (0.91 μg/mL) and CIP (1.32 μg/mL). Furthermore, the chromatograms obtained after DES^1^-MIR-SPE, DES^2^-MIR-SPE, DES^3^-MIR-SPE and MIR-SPE indicated that the interferences were all eliminated efficiently (Figure 7). On the other hand, the DES^1^-MIR-SPE method showed the highest recoveries of OFL (93.4%) and CIP (91.8%). These results show that the DES^1^-MIR-based SPE method will be promising for the routine monitoring of trace OFL and CIP in wastewater samples.

## 4. Conclusions

A novel water-compatible molecular imprinted resin was synthesized using DES as a green reaction solvent, resorcinol and melamine, as a double functional hydrophilic monomer and OFL as a template. Three types of DESs were selected to access the adsorption behavior. DES^1^ showed the best performance. Furthermore, DES^1^-MIR showed the special molecular recognition to the structural analogues of the template (OFL and CIP) in the aqueous matrices. The eco-friendly DES^1^-MIR was applied successfully as an SPE adsorbent for the extraction of OFL and CIP from wastewater and showed excellent recoveries and purification efficiency.

## Figures and Tables

**Table 1:** The details of synthesized hydrophilic deep eutectic solvents (DESs).

Abbreviation	HBA	HBD	Mole Ratio	Aspect
DES^1^	ChCl 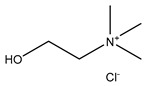	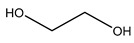 EG	HBA:HBD 1:2	Liquid
DES^2^	TMAB 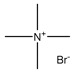			Liquid
DES^3^	TMAC 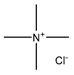			Liquid

**Table 2:** Synthesis scheme of DES-MIR.

Materials	#1: Monomer^1^ (mmol)	Crosslinking (mmol)	Solvent^2^ (mL)	#2: Monomer^2^ (mmol)	Crosslinking (mmol)	Solvent^2^ (mL)	Template (mmol)
MIR	30	60	MeOH 10	10	30	MeOH 20	0.25
DES^1^-MIR	30	60	DES^1^ 6	10	30	DES^1^ 6	0.25
DES^2^-MIR	30	60	DES^2^ 6	10	30	DES^2^ 6	0.25
DES^3^-MIR	30	60	DES^3^ 6	10	30	DES^3^ 6	0.25
NIR	30	60	MeOH 10	10	30	MeOH 10	-
DES^1^-NIR	30	60	DES^1^ 6	10	30	DES^1^ 6	-
DES^2^-NIR	30	60	DES^2^ 6	10	30	DES^2^ 6	-
DES^3^-NIR	30	60	DES^3^ 6	10	30	DES^3^ 6	-

#1: bottle #1; #2: bottle #2; Monomer^1^: resorcinol; Monomer^2^: melamine; Crosslinking: formaldehyde; Solvent^2^: MeOH, DES^1^, DES^2^ and DES^3^; Template: OFL.

**Table 3:** Isotherm model parameters for MIR, DES^1^-MIR, DES^2^-MIR and DES^3^-MIR on OFL adsorption.

Isotherm Model	Parameter	MIR	NIR	DES^1^-MIR	DES^2^-MIR	DES^3^-MIR
Langmuir	*R* ^2^	0.9786	0.9923	0.9618	0.9824	0.9840
	*Q*_max_	15.33	6.85	32.92	26.39	23.79
	*K*	0.027	0.058	0.047	0.047	0.042
Freundlich	*R* ^2^	0.9736	0.9373	0.9236	0.9663	0.9569
	*K*	1.31	1.34	3.63	3.30	2.60
	1/n	0.45	0.31	0.44	0.41	0.43
Scatchard	*R* ^2^	0.8334	0.9781	0.5652	0.8215	0.8245
	*Q*_max_	0.60	0.38	3.55	1.50	1.03
	*K*	−0.044	−0.056	−0.13	−0.061	−0.044

**Table 4:** Parameters of OFL adsorption towards DES^1^-MIR and MIR from two kinetic models.

Kinetic Model	Parameters	DES^1^-MIR	MIR
Pseudo-first-order	*R* ^2^	0.9911	0.8689
	*Q*_e_	27.31	4.21
	*K*_1_	0.020	0.018
Pseudo-second-order	*R* ^2^	0.9814	0.9972
	*Q*_e_	34.22	7.14
	*K*_2_	0.00034	0.0084

## Supplementary Materials

The following are available online at https://www.mdpi.com/2073-4360/11/5/871/s1, Table S1: Properties of the adsorbents, Table S2: Calibration equation, linear ranges, LOD and LOQ for the OFL and CIP with DES^1^-MIR-SPE method, Table S3: Intra-day and inter-day precision, accuracy and recovery of OLF and CIP at three different concentrations, Table S4: Comparison of the present method with previously reported methods, Table S5: Extraction and determination of OLF and CIP in real water sample from local environment with DES^1^-MIR-SPE method (n = 3).
