# Supplementary material for: Fabrication of Water-Compatible Molecularly Imprinted Resin in a Hydrophilic Deep Eutectic Solvent for the Determination and Purification of Quinolones in Wastewaters
Source: Polymers (Basel). 2019 May 13;11(5):871. doi: 10.3390/polym11050871 (PMC6571955; doi:10.3390/polym11050871)
Supplement: Supplementary file 1 [file polymers-11-00871-s001.pdf]

polymers

Supplementary Materials: Fabrication of Water-Compatible Molecularly Imprinted Resin in a Hydrophilic Deep Eutectic Solvent for the Determination and Purification of Quinolones in Wastewaters

Weiyang Tang and Kyung Ho Row

1. HPLC Conditions

HPLC was performed using YL9110 equipment from Younglin Co. Ltd. (Daegu, Korea), which included a YL9110 quaternary pump, YL9101 vacuum degasser, YL9131 column compartment, and YL9120 ultraviolet-visible dual channel detector. The analysis was conducted on a Chromatography Data System Autochro-3000 (Younglin Co. Ltd., Daegu, Korea) using an Optima pak C_{18} column (5 μm, 250 × 4.6 mm, RS Tech Corporation, Daejeon, Korea). The mobile phase was a 0.02 mol/L TMAB-ACN-TFA (90:10:0.06, v/v/v) solution with a flow rate of 1.0 mL/min. The injection volume was 10μL and the detection wavelength was set to 280 nm. The column temperature was set to 25 °C.

2. Synthesis of DES-based MIR

First, a 30 mmol resorcinol and 60 mmol formaldehyde solution (37%) was added to 6 mL of the DES (DES-1, DES-2, or DES-3) as the reaction solvent at 40 °C with stirring (bottle #1). Subsequently, 10 mmol melamine and 30 mmol formaldehyde were dissolved in 6 mL of DES and stirred at 80 °C until the solution became transparent (bottle #2). Bottle #2 was then allowed to cool to room temperature. The solution in bottle #2 was poured into bottle #1 with stirring. The next step was achieved by adding 0.25 mmol template (OFL) into bottle #1 for self-assembly for 1h at 40 °C. Subsequently, bottle #1 was heated to 80 °C for 24 h. The resulting solid was collected by suction filtration and washed several times with ultrapure water to remove the un-reacted reagents and DES. The obtained solid particles were eluted with methanol-water-acetic acid (8/1/1, v/v/v) until the elution did not contain the template, which was then checked by HPLC. Finally, the DES-MIR was washed with methanol to neutrality and dried at 50 °C for 24 h

Table S1. Properties of the adsorbents.

Specimens	N content (wt%)	Br or Cl content (wt%)
MIR	12.48	-
DES[1]-MIR	11.04	14.40(Cl)
DES[2]-MIR	7.34	36.13(Br)
DES[3]-MIR	8.21	25.42(Cl)

Table S2. Calibration equation, linear ranges, LOD and LOQ for the OFL and CIP with DES[1]-MIR-SPE method.

Compound	Calibration equation (μg/mL)	Linear range (μg/mL)	LOD (μg/mL)	LOQ (μg/mL)	R^2
OFL	y = 56.3x + 13.8	0.1–100	0.012	0.040	0.9995
CIP	y = 48.5x + 8.2	0.1–100	0.018	0.060	0.9989

Table S3. Intra-day and inter-day precision, accuracy and recovery of OLF and CIP at three different concentrations (*n* = 3) with DES[1]-MIR-SPE method.

Analyts	Spiked (μg/mL)	Intra-day		Inter-day		Method Recovery(%)
		Recovery (%)	RSD (%)	Recovery (%)	RSD (%)	-
OLF	1	93.4	3.3	91.7	3.1	92.3
	10	94.5	2.4	93.2	2.4	93.9
	100	93.5	1.9	94.5	2.2	94.0
CIP	1	89.2	3.7	91.5	4.6	90.4
	10	92.6	2.5	94.4	3.4	93.5
	100	91.4	2.1	88.7	3.0	90.1

Table S4. Comparison of the present method with previously reported methods.

Adsorbents	Sample	Linearity (μg/mL)	Recovery (%)	LOD (μg/mL)	RSD (%)	Ref.
MIP	Serum	0.35–150	90.7-101.2	0.07	2.9-4.1	Qiao et al[1]
Hybrid-MIP	Tilapia	0.05–25	87.3-100.6	0.0072	1.9-2.5	Yan et al[2]
Magnetic graphene	Food (Egg)	0.2–2	96.5-98.0	0.2	2.3-3.0	Wang et al[3]
MIP particle	Milk	0.02–1	78.3-97.7	0.01	2.4	Wang et al[4]
DES-MIR	Wastewater	0.1–100	90.1-94.0	0.012	1.9-4.6	This study

Table S5. Extraction and determination of OLF and CIP in real water sample from local environment with DES[1]-MIR-SPE method (n=3).

Sample	Analytes	Concentration before SPE (μg/mL)	Concentration after SPE (μg/mL)	Recovery (%)	RSD (%)
Sample[a]	OLF	*	*	*	*
	CIP	*	*	*	*
Sample[b]	OLF	0.35	*	93.0	2.6
	CIP	0.45	*	*	*
Sample[c]	OLF	0.91	0.850	93.4	2.0
	CIP	1.32	1.21	91.8	1.5

a: tap water; b: lake water; c: seafood market water; *: not detected.

References

1. Qiao, F.; Yan, H. Simultaneous analysis of fluoroquinolones and xanthine derivatives in serum by molecularly imprinted matrix solid-phase dispersion coupled with liquid chromatography. *J. Chromatogr.* B, **2011**, *879*, 3551–3555.
2. Yang, X.; Wang, R.; Wang, W.; Yan, H.; Qiu, M.; Song, Y. Synthesis of a novel molecularly imprinted organic–inorganic hybrid polymer for the selective isolation and determination of fluoroquinolones in tilapia. *J. Chromatogr. B* **2014**, *945*, 127–134.
3. He, X.; Wang, G.N.; Yang, K.; Liu, H.Z.; Wu, X.J.; Wang, J.P. Magnetic graphene dispersive solid phase extraction combining high performance liquid chromatography for determination of fluoroquinolones in foods. *Food Chem.* **2017**, *221*, 1226–1231.
4. Wang, G.N.; Yang, K.; Liu, H.Z.; Feng, M.X.; Wang, J.P. Molecularly imprinted polymer-based solid phase extraction combined high performance liquid chromatography for determination of fluoroquinolones in milk. *Anal. Methods* **2016**, *8*, 5511–5518.

© 2019 by the authors. Licensee MDPI, Basel, Switzerland. This article is an open access article distributed under the terms and conditions of the Creative Commons Attribution (CC BY) license (http://creativecommons.org/licenses/by/4.0/).
